# Internal carotid artery blood flow in healthy awake subjects is reduced by simulated hypovolemia and noninvasive mechanical ventilation

**DOI:** 10.14814/phy2.12969

**Published:** 2016-10-04

**Authors:** Maria Skytioti, Signe Søvik, Maja Elstad

**Affiliations:** ^1^ Division of PhysiologyInstitute of Basic Medical SciencesUniversity of OsloOsloNorway; ^2^ Deptartment of Anaesthesia and Intensive CareAkershus University HospitalLørenskogNorway

**Keywords:** Cerebral blood flow, cerebrovascular circulation, hypovolemia, internal carotid artery blood flow, noninvasive ventilation

## Abstract

Intact cerebral blood flow (CBF) is essential for cerebral metabolism and function, whereas hypoperfusion in relation to hypovolemia and hypocapnia can lead to severe cerebral damage. This study was designed to assess internal carotid artery blood flow (ICA‐BF) during simulated hypovolemia and noninvasive positive pressure ventilation (PPV) in young healthy humans. Beat‐by‐beat blood velocity (ICA and aorta) were measured by Doppler ultrasound during normovolemia and simulated hypovolemia (lower body negative pressure), with or without PPV in 15 awake subjects. Heart rate, plethysmographic finger arterial pressure, respiratory frequency, and end‐tidal CO_2_ (ETCO_2_) were also recorded. Cardiac index (CI) and ICA‐BF were calculated beat‐by‐beat. Medians and 95% confidence intervals and Wilcoxon signed rank test for paired samples were used to test the difference between conditions. Effects on ICA‐BF were modeled by linear mixed‐effects regression analysis. During spontaneous breathing, ICA‐BF was reduced from normovolemia (247, 202–284 mL/min) to hypovolemia (218, 194–271 mL/min). During combined PPV and hypovolemia, ICA‐BF decreased by 15% (200, 152–231 mL/min, *P* = 0.001). Regression analysis attributed this fall to concurrent reductions in CI (*β*: 43.2, SE: 17.1, *P* = 0.013) and ETCO_2_ (*β*: 32.8, SE: 9.3, *P* = 0.001). Mean arterial pressure was maintained and did not contribute to ICA‐BF variance. In healthy awake subjects, ICA‐BF was significantly reduced during simulated hypovolemia combined with noninvasive PPV. Reductions in CI and ETCO_2_ had additive effects on ICA‐BF reduction. In hypovolemic patients, even low‐pressure noninvasive ventilation may cause clinically relevant reductions in CBF, despite maintained arterial blood pressure.

## Introduction

Preserving cerebral blood flow (CBF) in patients during surgery and anesthesia may be challenging as it requires good control over both circulation and ventilation, and CBF is not easily monitored. Factors such as dehydration, acute blood loss or acute peripheral vasodilation that change intravascular volume may compromise cerebral perfusion. Baroreflex engagement and activation of the sympathetic nervous system (SNS) compensate for mild to moderate hypovolemia, maintaining mean arterial blood pressure (MAP). Lassen's CBF autoregulation curve is traditionally used to guide clinical decisions concerning safe blood pressure limits (Lassen 1959).

Cardiac output (CO) has been found to determine CBF independently of MAP. A linear relationship between middle cerebral artery blood velocity and CO has been demonstrated despite MAP preservation (Ogoh et al. 2005). In another study however beat‐to‐beat CO changes did not correspond with changes in CBF velocity following thigh‐cuff release and significant MAP reduction (Deegan et al. 2010b). Apart from circulatory factors, cerebrovasculature is highly reactive to the arterial partial pressure of carbon dioxide (PaCO_2_), and even mild hypocapnia can significantly reduce global CBF (Ide et al. 2003; Sato et al. 2012). However, regional differences in response to hypocapnia and hypotension have been reported between the intracranial and extracranial arteries as well as between anterior and posterior cerebral circulation (Lewis et al. 2015).

The respiratory pump, among others mechanisms, functions as an important counteracting mechanism in hypovolemia, as demonstrated in healthy volunteers (Convertino et al. 2011; Poh et al. 2014). The negative pressure generated during inspiration enhances venous return, preload and CO (Convertino et al. 2011). Respiratory pump function and resulting changes in intrathoracic pressure may have an important regulatory role on cerebral circulation as well (Yannopoulos et al. 2006; Lucas et al. 2013; Segal et al. 2013). Positive pressure ventilatory support of patients employed in anesthesia and intensive care abolishes the generation of negative intrathoracic pressure and reduces the beneficial circulatory effect of the respiratory pump (Cheifetz 2014). The effects of positive pressure ventilation (PPV) on venous return and preload are reported to be more pronounced during hypovolemic states (Cheifetz 2014).

The aim of this study was to investigate central circulatory and CBF changes in young healthy subjects during standardized challenges: simulated moderate hypovolemia and noninvasive PPV. We used a lower body negative pressure (LBNP) chamber to induce abrupt central blood volume shifts during spontaneous breathing and PPV. The application of −30 mmHg LBNP induces moderate hypovolemia corresponding to 10–20% of total blood volume (Cooke et al. 2004). We hypothesized the addition of PPV to hypovolemia would result in a greater drop in CBF. We also hypothesized that CBF changes during PPV and/or LBNP could be explained by changes in end‐tidal CO_2_ (ETCO_2_), CO, and MAP.

## Materials and Methods

### Subjects

Fifteen young healthy volunteers, seven males and eight females, age 22 (median, range: 20–30) years, body mass index 23.4 (median, range: 18.0–26.7) were recruited and gave written, informed consent to participate. All procedures conformed to the Declaration of Helsinki. The regional ethics committee (ref.no: 2014/2228, January 2015) approved the protocol and procedures.

None of the subjects were tobacco smokers or taking any medication. They were instructed to abstain from caffeine and strenuous physical activity for at least 12 h and avoid food and drink for 2 h and alcohol for 24 h before each experiment. All subjects reported good health and hydration status on the day of the experiment.

### Experimental protocol

Experiments took place in a quiet room with an ambient temperature of 22–24°C, continuously recorded to ensure stable conditions. All experiments took place between 11.00 am and 3.00 pm. Pressure‐regulated volume control ventilation (VIVO50, Diacor a/s, Norway) was administered noninvasively via a face mask (Respireo Primo F Non Vented, Air Liquide Medical Systems, Italy). A customized LBNP chamber was used to induce simulated hypovolemia (Hisdal et al. 2003). The subjects were trained not to initiate inspiration but to breathe in synchrony with the ventilator. Ventilator settings (respiratory frequency [RF] and tidal volume [TV]) were chosen to just override each subject's spontaneous respiratory pattern. Set values (median [range]) were as follows: inspiration time: 1.25 sec (1.2–1.4); RF: 14 breaths per min (Eriksen and Walloe 1990; Fu et al. 2004, 2005; Deegan et al. 2010b; Dippmann et al. 2010; Fitzmaurice et al. 2011); target TV: 650 mL (500–850). Maximum and minimum inspiratory pressures were set to 14 cmH_2_O and 4.5 cmH_2_O, respectively, for all subjects. No inspiratory trigger was used. Positive end‐expiratory pressure (PEEP) was set to 1 or 2 cmH_2_O. Table 1 presents medians and 95% Confidence Intervals (95% CI) of recorded peak inspiratory pressure (Ppeak), mean inspiratory pressure (Pmean), PEEP, and TV. The subjects visited the laboratory on 1 day for acclimatization, and on a second day for the recordings.

**Table 1:** Ventilator readings during noninvasive PPV, with and without simulated hypovolemia

	Positive pressure ventilation (PPV)	
	Normovolemia	Hypovolemia
Ppeak (cmH_2_O)	8.8 (7.8, 9.6)	8.2 (6.9, 9.2)
Pmean (cmH_2_O)	3.5 (3.15, 3.7)	3.4 (3.0, 3.5)
PEEP (cmH_2_O)	1.3 (0.9, 1.4)	1.2 (0.8, 1.4)
TV (mL)	659 (610, 694)	662 (605, 741)

Data are presented as medians and 95% confidence intervals calculated by Hodges–Lehmann's estimate. Ppeak, peak airway pressure; Pmean, mean airway pressure; PEEP, positive end expiratory pressure; TV, tidal volume. (*n* = 12).

Before the experiment, the diameters of the subject's right internal carotid artery (ICA) and rigid aortic ring were obtained at the measurement site using B‐mode Ultrasound (10 MHz and 2.5 MHz, System Five, GE Vingmed Sound, Horten, Norway) (Eriksen and Walloe 1990).

**Figure 1:**
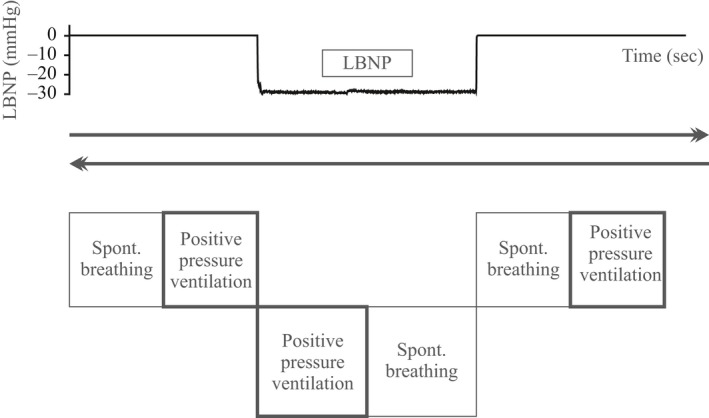
Study protocol displaying subjects' breathing conditions and lower body chamber pressure during 10 min of normovolemia, 10 min of simulated hypovolemia and 10 min of normovolemia–recovery. Arrows indicate that the sequence was run twice in each subject, once starting with noninvasive positive pressure ventilation and once with spontaneous breathing. LBNP, lower body negative pressure.

The subjects lay supine in the LBNP chamber with the face mask throughout the procedure. This started with a 10‐min baseline period of normovolemia. Simulated central hypovolemia was then induced abruptly (within 0.3 sec) (Hisdal et al. 2003) by −30 mmHg LBNP and maintained for the next 10 min. The procedure ended with a 10‐min recovery period of normovolemia. During each 10‐min period, the subjects breathed spontaneously for 5 min and were subjected to volume‐controlled PPV for 5 min (Fig. 1). The procedure was run twice for each subject, with a 5‐min pause between rounds. The first 30‐min round was randomized to start with either PPV or spontaneous breathing; in the second round the sequence was reversed.

The experiment was to be terminated immediately in the event of a drop in MAP > 15 mmHg, a drop in heart rate (HR) > 15 bpm, systolic blood pressure <70 mmHg, or if the subject suffered presyncopal symptoms or simply wanted the procedure to end (Cooke et al. 2004).

### Recordings

Mean ICA velocity (5 MHz probe, insonation angle: 45°, SD‐100, Vingmed Sound) and maximum aortic velocity (2 MHz probe, insonation angle: 20°, SD‐50, Vingmed Sound) were recorded concurrently by two trained operators, using Doppler ultrasound. ICA velocity was measured approximately 2 cm above the bifurcation of the common carotid artery in order to avoid turbulent flow (Willie et al. 2012). Aortic velocity was recorded from the suprasternal notch. Finger arterial pressure was recorded continuously from the middle left finger (Finometer, Finapres Medical System, the Netherlands) positioned at heart level, and beat‐by‐beat MAP was calculated by numerical integration. The Finometer also provided pulse rate and cardiac stroke volume (SV) estimates. RF was recorded using an elastic belt around the abdomen (Respiration and body position amplifier, Scan‐Med a/s, Norway). HR was calculated from the R‐R distance in a three‐lead ECG. Expiratory CO_2_ was sampled continuously from the face mask near the nares, recorded by infrared spectroscopy (Artema MM201, Artema Medical AB, Sweden) and calculated into ETCO_2_ breath‐by‐breath. Blood velocity waves, RF, ECG, arterial blood pressure waves, HR (finometer), SV (finometer), LBNP, and room temperature were sampled at 100 Hz and transferred on‐line to a recording computer running a dedicated data collection and analysis program (program for real‐time data acquisition, Morten Eriksen, Norway).

SV and ICA beat volume (ICA‐BV) were calculated from blood velocities and the diameters of the aortic valvular orifice (Eriksen and Walloe 1990) and ICA, respectively. In addition, CO and ICA blood flow (ICA‐BF) were calculated beat‐by‐beat from SV and ICA‐BV, respectively, multiplied by instantaneous HR. Cardiac index (CI) was calculated by dividing CO by body surface area.

### Signal processing and statistical analysis

All datasets were resampled at 4 Hz and analyzed by a custom‐made program written in MATLAB (version R2013b, Mathworks, Natick, MA). The recorded signals were inspected and medians were calculated along 2‐min periods of continuous, technically successful recordings from each 5‐min period. All technically successful recordings from each subject were included in the analysis. We compared the cardiovascular values in four different states: Spontaneous breathing during normovolemia (Normovolemia), positive pressure ventilation during normovolemia (PPV), Spontaneous breathing and hypovolemia (Hypovolemia) and PPV and hypovolemia combined (PPV + hypovolemia).

Medians and 95% CI were calculated by Hodges–Lehmann's estimate (Hollander and Wolfe 1999) and the Wilcoxon matched‐pairs signed rank test against a two‐sided alternative (Hollander and Wolfe 1999) was used to test differences between states (StatXact, Cytel Studio 10, Cytel Inc., Cambridge, MA, USA). ICA‐BF and ICA‐BV in hypovolemia with and without PPV were compared to normovolemic levels. Because multiple comparisons were used, the significance level was Bonferroni‐corrected and set at *P* = 0.01.

The ICA‐BF response to PPV and/or simulated hypovolemia was modeled using linear mixed‐effects multiple regression (SAS‐JMP 12 software for Windows, SAS Institute, Cary, NC). ICA‐BF was the response variable. LBNP and PPV (dichotomous variables: Yes/No) and the randomized initial stimulus of the sequence (dichotomous variable: PPV/Spontaneous Breathing) were categorical predictors (fixed effects). CI, MAP, ETCO_2_, and Pmean were continuous predictors (covariates). Because the study used a repeated‐measures design, each subject contributed minimum 3 and maximum 12 observations. In order to account for the correlation among the repeated observations in each subject, Subject identity was entered as random effect in the model.

Linear mixed‐effects models can handle unequal numbers of observations among subjects and account for the correlation between repeated observations from the same subject (Fitzmaurice et al. 2011). Both fixed and random effects were specified for a standard least squares fit using the restricted maximum likelihood method (Searle et al. 2008) for the estimation of fixed effects coefficients and variance component estimates for random effects; the covariance structure used was the Variance Component structure. A backwards variable selection was performed by initially entering all the mentioned explanatory variables in the model and removing them one by one, starting with the least significant. Variables with a *P* < 0.1 were kept in the model as a variable marginally associated with the outcome may potentially have a considerable effect on the coefficients of the other explanatory variables (Katz 2006). Inspection of the residual plot revealed no deviations from the assumptions of normality and homoscedasticity.

To assess the goodness of fit, the RSquared (or coefficient of multiple determination) was calculated using quantities from the corresponding Analysis of Variance table.

The *P*‐values were obtained by likelihood ratio tests of the full model with the relevant effects against the model without these effects (null model). The statistical significance level was set at *P* < 0.05.

## Results

ICA velocity recordings were analyzed from 15 subjects in total. More specifically, in ten subjects we obtained good ICA velocity recordings from both rounds; in two subjects only recordings from one round were of adequate quality to be included in the analysis. The last three subjects were unable to breathe in synchrony with the ventilator and thus provided only partial data (without PPV). In six of 15 subjects, recordings of aorta velocities were of inadequate quality, an anticipated failure rate. CO from the noninvasive arterial blood pressure curve was obtained in all subjects.

**Figure 2:**
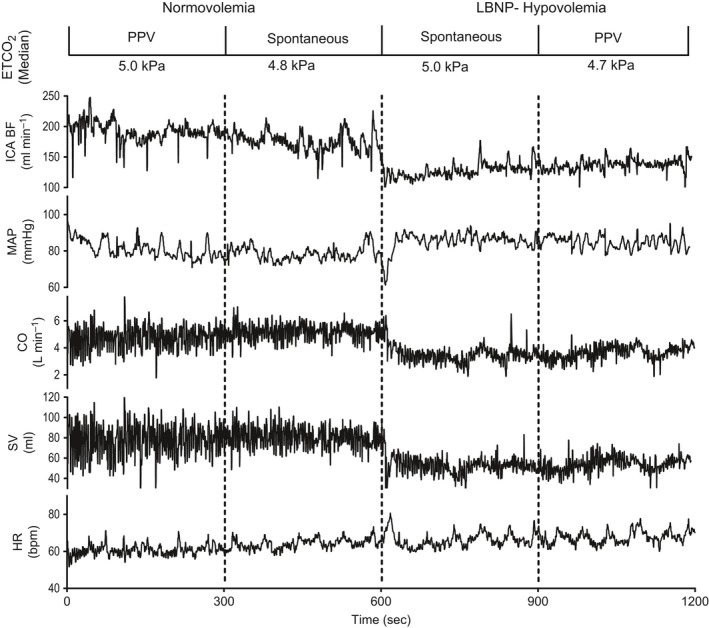
Recordings of internal carotid artery blood flow (ICA‐BF), mean arterial pressure (MAP), cardiac output (CO), stroke volume (SV), and heart rate (HR) in one subject during 10 min of normovolemia and 10 min of hypovolemia. ETCO_2_, end‐tidal CO_2_.

Induction of LBNP (central hypovolemia) caused a drop in ICA‐BF and a simultaneous drop in CI and SV. A concurrent, transient drop in MAP was rapidly restored, while HR increased during hypovolemia (Fig. 2). The changes in cardiovascular and respiratory variables during the different experimental states are presented in Table 2.

**Table 2:** Changes in cardiovascular and respiratory variables during different experimental conditions

	Normovolemia		Hypovolemia	
	Spontaneous breathing	PPV	Spontaneous breathing	PPV
ICA‐BF (mL/min)	247 (202, 284)	213^a^ (158, 241) *n* = 12	218 (194, 271)	200^a^ (152, 231) *n* = 12
ICA‐BV (mL)	4.6 (3.4, 5.0)	4.1^a^ (2.7, 4.5) *n* = 12	3.7^a^ (3.0, 4.1)	3.2^a^ (2.3, 3.7) *n* = 12
CO (us, L/min)	4.6 (4.0, 4.9) *n* = 11	4.6 (3.4, 4.9) *n* = 9	3.6^a^ (2.9, 3.8) *n* = 11	3.4^a^ (2.6, 3.6) *n* = 9
CI (us, L/min/m^2^)	2.6 (2.4, 2.7) *n* = 11	2.6 (1.9, 2,8) *n* = 9	2.0^a^ (1.6, 2.2) *n* = 11	1.9^a^ (1.5, 2.0) *n* = 9
SV (us, mL)	80.4 (72.5, 84.4) *n* = 11	77.9 (64.6, 86.9) *n* = 9	55.1^a^ (45.4, 58.8) *n* = 11	48.6^a^ (38.7, 52.9) *n* = 9
CO (finometer, L/min)	5.1 (4.0, 5.5)	5.0 (3.7, 5.5) *n* = 12	4.5^a^ (3.7, 5.0)	4.4^a^ (3.2, 4.9) *n* = 12
SV (finometer, mL)	87.4 (74.6, 91.3)	89.8 (73.1, 91.7) *n* = 12	68.7^a^ (58.7, 75.0)	65.6^a^ (52.4, 72.5) *n* = 12
HR (bpm)	58.0 (51.6, 60.2)	55.9 (48.9, 58.8) *n* = 12	63.8^a^ (58, 66.8)	64.4^a^ (57.4, 66.5) *n* = 12
MAP (mmHg)	75.8 (70.2, 78.8)	77.2 (71.3, 79.9) *n* = 12	78.7^a^ (73.4, 81.7)	79.0 (74.2, 81.8) *n* = 12
ETCO_2_ (kPa)	4.7 (4.4, 4.9)	4.3^a^ (3.9, 4.6) *n* = 12	4.6 (4.2, 4.7)	3.9^a^ (3.5, 4.1) *n* = 12
RF (breaths per min)	13.9 (12.3, 15.2)	13.8 (12.9, 14.4)	12.5 (11.0, 13.1)	13.8 (12.6, 14.4)

Data are presented as medians and 95% confidence intervals calculated by Hodges–Lehmann's estimate. PPV, positive pressure ventilation; ICA, internal carotid artery; BF, blood flow, BV, beat volume, CO, cardiac output; CI cardiac index; SV, stroke volume; HR, heart rate; bpm, beats per minute; MAP, mean arterial pressure; EtCO_2_, end‐tidal CO_2_; RF, respiratory frequency; us, ultrasound (*n* = 15 unless reported otherwise).

a *P* < 0.01 with pairwise Wilcoxon signed rank test comparisons with normovolemia + spontaneous breathing.

### Effect of hypovolemia on cardiovascular variables

ICA‐BF tended to decrease with a median change of 16 mL/min (−7.0–30.0, −6%, *P* = 0.15) from normovolemia to hypovolemia, whereas ICA‐BV significantly decreased by 0.8 mL (0.3–1.1, −17%, *P* = 0.002). CI was reduced by 0.7 L/min/m^2^ (0.4–0.8, −27%) from normovolemia to hypovolemia. MAP showed an increase of 3.2 mmHg (1.1–4.1) and HR increased by 6 bpm (3.7–7.9) from normovolemia to hypovolemia.

### Effect of PPV on cardiovascular variables

ICA‐BF showed a median decrease of 29 mL/min (19–49, −11%, *P* = 0.003) from normovolemia to PPV. ICA‐BV decreased by 0.4 mL (0.3–0.8, −9%, *P* = 0.007). No change was observed between normovolemia and PPV in CI and MAP. HR tended to decrease by 1.4 bpm (0.8–2.8, *P* = 0.02).

### Effect of PPV + hypovolemia on cardiovascular variables

ICA‐BF decreased significantly by 37 mL/min (17–48, −15%, *P* = 0.001) from normovolemia to PPV + hypovolemia. ICA‐BV decreased by 1.1 mL (0.6–1.5, −24%, *P* < 0.001). CI was reduced by 0.8 L/min/m^2^ (0.6–0.9, −30%) from normovolemia to PPV + hypovolemia. MAP showed no change to PPV + hypovolemia (1.1 mmHg (−2.0–3.0)) and HR increased by 7 bpm (3.6–10.2).

### ETCO_2_ changes during hypovolemia and PPV

ETCO_2_ was preserved from normovolemia to hypovolemia (0.2 kPa (0–0.3), Table 2). It decreased from normovolemia to PPV (0.4 kPa (0.1–0.6)) and from normovolemia to PPV + hypovolemia (0.8 kPa (0.5–1.1)), resulting in mild hypocapnia (Table 2). Changes in RF were marginal, less than 2 breaths per min.

### ICA‐BF prediction model

The linear mixed‐effects model could explain 78% of the variance in ICA‐BF (Adjusted *R* ^2^ = 0.78, *P* = 0.0001). CI and ETCO_2_ contributed significantly to ICA‐BF variance; MAP, Pmean and PPV did not, and were removed from the model. A drop of 1 L/min/m^2^ in CI predicted a 43 mL/min drop in ICA‐BF (*P* = 0.013), whereas a drop of 1 kPa in ETCO_2_ predicted a drop of 33 mL/min in ICA‐BF (*P* = 0.001, Table 3). Which stimulus the experimental sequence started with (PPV vs. spontaneous breathing) also contributed slightly (less than 10 mL/min) to ICA‐BF variance (Table 3). No more‐than‐additive effect of LBNP and PPV on ICA‐BF response could be demonstrated. Of the total random variance, 67% was attributed to variability between subjects.

**Table 3:** Estimated regression coefficients (fixed effects) and standard errors for internal carotid artery blood flow response

	*β*	SE	*t* ratio	*P*‐value
ETCO_2_ (kPa)	32.8	9.3	3.53	0.001^a^
CI (L/min/m^2^)	42.6	17.1	2.49	0.013^a^
Start stimulus (PPV vs. SPONT)	9.5	4.4	2.16	0.033^a^
LBNP(Yes vs. No)	−12.7	7.0	−1.82	0.072

*β*, regression coefficient; SE, standard error; ETCO_2_, end‐tidal CO_2_; CI, cardiac index; LBNP, lower body negative pressure; PPV, positive pressure ventilation; SPONT, spontaneous breathing.

a Statistically significant, *P* < 0.05.

This analysis was also performed using ICA velocity as the response variable, since blood velocity is often used as a substitute for blood flow. The second analysis showed that ICA velocity was predicted by CI (*P* = 0.001) and PPV (*P* = 0.003).

## Discussion

We induced abrupt hypovolemia in an awake, spontaneously breathing or noninvasively ventilated subject. The combination of hypovolemia and PPV was associated with a 15% reduction in ICA‐BF, whereas MAP was stable during compensated central hypovolemia. CI and ETCO_2_ contributed significantly to these changes. Our results suggest that CBF is vulnerable to hypovolemia and mild hypocapnia despite circulatory reflex adaptations and absence of hypotension.

On the induction of hypovolemia, MAP decreased transiently, being restored after 20 sec despite the large persisting reduction in SV and CI. Baroreceptor unloading and SNS activation probably mediated MAP restoration and HR increase. Despite the restored MAP, the reduction in CI was a significant predictor of ICA‐BF drop in our model, analogous to previously reported results (Ogoh et al. 2005; Meng et al. 2015). In a recent review, the integrated effect of the various CBF‐regulatory mechanisms, including CO, on the cerebrovascular resistance and the dynamic nature of the cerebral autoregulation curve are discussed; a downwards shift of the autoregulatory plateau during CO reduction is suggested (Meng et al. 2015).

The slight increase in HR during hypovolemia ameliorated the drop in ICA‐BF (15%) when ICA‐BV was decreased by 24%. Thus, despite the counteracting circulatory adaptations to simulated hypovolemia, ICA‐BF decreased significantly under both LBNP and PPV due to a drop in CI combined with mild hypocapnia.

The drop in CI may have decreased peripheral tissue perfusion and/or metabolism, reducing CO_2_ transport to the lungs. The hypocapnic reactivity of CBF has been calculated to 2–3%/mmHg (Sato et al. 2012). Thus, the observed 0.8 kPa (0.5–1.1) drop in ETCO_2_ would predict a 12–18% reduction in ICA‐BF. We observed a 15% reduction in ICA‐BF and a 24% reduction in ICA‐BV during the combined effect of hypovolemia and mild hypocapnia.

We chose to study ICA‐BF as 65–70% of global CBF is supplied by ICAs (Schoning et al. 1994). Concerning regional differences in cerebral circulation, a steady decrease in ICA‐BF has been reported during graded LBNP while vertebral artery blood flow remained unchanged and MAP was maintained (Ogoh et al. 2015). In contrast, Lewis et al. (2015) report a decline in ICA‐BF and vertebral artery blood flow during LBNP accompanied by a reduction of 20% in MAP; the decline in vertebral artery blood flow was significantly less with prevention of hypocapnia. Deegan et al. (2010a) report a similar response between vertebral and middle cerebral arteries 5 min before presyncope during orthostatic stress. Consequently, anterior and posterior cerebral circulations may respond differently to graded orthostatic challenges.

An interesting consideration is whether SNS activation due to LBNP contributed to the drop in ICA‐BF through sympathetic mediated cerebral vasoconstriction (Levine et al. 1994; Ainslie et al. 2005; Meng et al. 2015). It has been reported that gradual LBNP led to a decrease in CBF velocity before the appearance of hypotension, accompanied by increased pulsatility as an indication of cerebral vasoconstriction (Levine et al. 1994). In another study where autonomic ganglionic blockade was used, the drop in CBF during moderate LBNP could not be attributed primarily to cerebral vasoconstriction (Zhang and Levine 2007). Recent work has demonstrated that muscle sympathetic nervous activity activation is linearly and inversely related to SV reduction, indicating that SV and CO are important contributors to arterial baroreflex regulation even if MAP is preserved (Charkoudian et al. 2005; Fu et al. 2005).

Pmean and PPV did not contribute to ICA‐BF variance in our model. This was probably due to the low‐grade positive intrathoracic pressure inflicted, the lack of Pmean measurements during spontaneous breathing, and the close correlation between PPV and slight hypocapnia, making it difficult to statistically distinguish between their effects. When ICA blood velocity was used as the response variable, the dichotomous variable PPV (Yes/No) contributed to ICA blood velocity variance while ETCO_2_ did not, indicating that intrathoracic pressure (positive/negative) may influence ICA‐BF regulation.

Interestingly, the respiratory state starting the randomized sequence (PPV or spontaneous breathing) was found to contribute slightly but significantly to ICA‐BF variance. Within‐experiment “learning” has been demonstrated for other cardiorespiratory control systems, but our data cannot point to any underlying mechanisms.

Gender differences in response to LBNP have been reported regarding several determinants of CBF such as SV, CO, and sympathetic nerve activity. Women are considered to be less tolerant to hypovolemia at presyncopal level (Convertino 1998; Fu et al. 2004; Yang et al. 2012). In this study, we induced mild to moderate compensated hypovolemia with a low degree of LBNP (−30 mmHg) and none of our subjects experienced hypotension or presyncopal symptoms. The changes in SV, CO, and ICA‐BF from normovolemia to hypovolemia were similar between males and females. However, our study was not powered to look at gender differences.

In clinical situations, the combination of volume depletion and mild hypocapnia can be a threat to cerebral perfusion (Dippmann et al. 2010; Lee et al. 2011; Jeong et al. 2012). Even our healthy, young, unsedated subjects, supposedly with intact counteracting circulatory adaptive mechanisms, showed a significant ICA‐BF decrease of 15% during central hypovolemia and low‐pressure mechanical ventilation. Without the reflex tachycardia during hypovolemia, the drop would have been about 24%, clinically relevant in patients with a poor chronotropic response. Standard clinical HR and MAP monitoring would not have revealed the drop in CI, no presyncopal symptoms occurred, and a mild hypocapnia is likely to go undetected in a clinical setting. Importantly, ICA‐BF was better preserved during hypovolemia when the subjects breathed spontaneously (Poh et al. 2014).

Our experiments most closely resembled an intensive care unit situation where a hypovolemic unsedated patients is treated with noninvasive ventilation. Older age, comorbidities, anesthetic agents, invasive ventilatory support or the need for higher intrathoracic pressures could aggravate the situation further, as would acute blood loss, dehydration, and pneumoperitoneum during laparoscopy.

**Figure 3:**
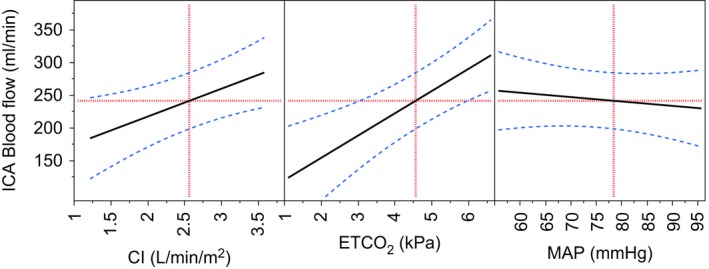
Predicted ICA‐BF response to changes in cardiac index (CI), ETCO_2_, and mean arterial pressure (MAP). Solid line: mean ICA‐BF response. Blue dotted lines: 95% confidence intervals. Horizontal red dotted line: ICA‐BF response at given CI, ETCO_2_, and MAP. Vertical red dotted lines: values of CI, ETCO_2_ and MAP. ICA‐BF, internal carotid artery blood flow; PPV, positive pressure ventilation; LBNP, lower body negative pressure; ETCO_2_, end‐tidal carbon dioxide.

Our findings confirm and supplement previous findings indicating that preservation of MAP in the “plateau area” of the well‐known cerebral autoregulation curve may not be sufficient to preserve cerebral perfusion. Lassen's curve of cerebral autoregulation was constructed by data points obtained from different patient groups with hypotensive or hypertensive disorders. This method generated a group curve with a wide “plateau area” with a lower limit, a curve that cannot precisely predict the CBF–MAP relationship in individual subjects (Tzeng and Ainslie 2014). In normotensive patients, blood flow to the brain may still be compromised by changes in CI and ETCO_2_ (Meng and Gelb 2015; Meng et al. 2015). In cases of noncompensated hypovolemia and in patients unable to increase HR due to autonomic dysfunction, medication, or anesthetic agents, CBF may be further compromised. Our findings suggest that in a hypothetical clinical situation of compensated hypovolemia, slight permissive hypercapnia may improve blood flow to the brain (Fig. 3). On the other hand, there is evidence that dynamic cerebral autoregulation is improved by hypocapnia in relation to normocapnia and hypercapnia (Querido et al. 2013). Prevention of hypercapnia or slight hypocapnia could therefore protect cerebral perfusion during unstable circulatory states.

### Methodological considerations – limitations

In healthy humans with no significant ventilation–perfusion mismatch, the usual difference between PaCO_2_ and ETCO_2_ values is 0.27–0.67 kPa (Anderson and Breen 2000). A decrease in CI may slightly increase this alveolar‐arterial difference. Thus, the demonstrated effect of ETCO_2_ on ICA‐BF may be slightly overestimated. ETCO_2_ provides an accurate estimate of PaCO_2_ in mechanically ventilated and nonintubated patients (Barton and Wang 1994; Casati et al. 2001; Takano et al. 2003).

To calculate ICA‐BF, we assumed that vessel diameter remained stable throughout the experiment. It is debated whether PaCO_2_ changes >1.3 kPa cause ICA diameter changes (Sato et al. 2012; Willie et al. 2012). A 5% decline in ICA diameter has also been reported during a 20% MAP reduction induced by LBNP (Lewis et al. 2015). No change in ICA diameter is reported by Ogoh et al. (2015) between baseline and −35 mmHg of LBNP. As the largest change in ETCO_2_ in our experiments was 1.1 kPa, MAP was preserved and the LBNP level was −30 mmHg, we assumed that ICA diameter remained constant.

For simplicity, we used a linear approach to model ICA‐BF changes, since an almost linear relationship has been reported in the range 3.3–7.3 kPa (Hauge et al. 1980). Unlike other studies using velocity instead of flow, our model did not reveal a significant relationship between ICA velocity and ETCO_2_ when PPV was included in the model, probably because these variables were very closely correlated. With PPV removed, ETCO_2_ was found to contribute significantly to ICA velocity variance.

## Conclusions

The combination of simulated moderate hypovolemia and noninvasive mechanical ventilation resulted in a significant decrease in ICA‐BF, larger than in PPV or hypovolemia alone. The drop in ICA‐BF was mediated by slight hypocapnia and a marked fall in CI and occurred despite an unchanged MAP. Our findings indicate that in a clinical setting of hypovolemia close attention to CO measurements and ventilator settings is needed to preserve patient CBF.

## Conflict of Interest

None declared.
